# Real-life data on monoclonal antibodies and antiviral drugs in Italian inborn errors of immunity patients during COVID-19 pandemic

**DOI:** 10.3389/fimmu.2022.947174

**Published:** 2022-07-28

**Authors:** Giulia Garzi, Francesco Cinetto, Davide Firinu, Giulia Di Napoli, Gianluca Lagnese, Alessandra Punziano, Patrick Bez, Bianca Laura Cinicola, Giulia Costanzo, Riccardo Scarpa, Federica Pulvirenti, Marcello Rattazzi, Giuseppe Spadaro, Isabella Quinti, Cinzia Milito

**Affiliations:** ^1^ Department of Molecular Medicine, Sapienza University of Rome, Rome, Italy; ^2^ Department of Medicine—DIMED, University of Padova, Padua, Italy; ^3^ Rare Diseases Referral Center, Internal Medicine I, Ca’ Foncello Hospital, AULSS2 Marca Trevigiana, Treviso, Italy; ^4^ Department of Medical Sciences and Public Health, University of Cagliari, Monserrato, Italy; ^5^ Department of Translational Medical Sciences, University of Naples Federico II, Naples, Italy; ^6^ Department of Maternal Infantile and Urological Sciences, Sapienza University of Rome, Rome, Italy; ^7^ Regional Reference Centre for Primary Immune Deficiencies, Azienda Ospedaliera Universitaria Policlinico Umberto I, Rome, Italy

**Keywords:** inborn errors of immunity, COVID-19, monoclonal antibodies, antiviral drugs, hospitalization-risk, disease severity, duration of viral shedding, reinfection

## Abstract

**Background:**

Since the beginning of the COVID-19 pandemic, patients with Inborn Errors of Immunity have been infected by SARS-CoV-2 virus showing a spectrum of disease ranging from asymptomatic to severe COVID-19. A fair number of patients did not respond adequately to SARS-CoV-2 vaccinations, thus early therapeutic or prophylactic measures were needed to prevent severe or fatal course or COVID-19 and to reduce the burden of hospitalizations.

**Methods:**

Longitudinal, multicentric study on patients with Inborn Errors of Immunity immunized with mRNA vaccines treated with monoclonal antibodies and/or antiviral agents at the first infection and at reinfection by SARS-CoV-2. Analyses of efficacy were performed according to the different circulating SARS-CoV-2 strains.

**Results:**

The analysis of the cohort of 192 SARS-CoV-2 infected patients, across 26 months, showed the efficacy of antivirals on the risk of hospitalization, while mabs offered a positive effect on hospitalization, and COVID-19 severity. This protection was consistent across the alpha, delta and early omicron waves, although the emergence of BA.2 reduced the effect of available mabs. Hospitalized patients treated with mabs and antivirals had a lower risk of ICU admission. We reported 16 re-infections with a length of SARS-CoV-2 positivity at second infection shorter among patients treated with mabs. Treatment with antivirals and mabs was safe.

**Conclusions:**

The widespread use of specific therapy, vaccination and better access to care might have contributed to mitigate risk of mortality, hospital admission, and severe disease. However, the rapid spread of new viral strains underlines that mabs and antiviral beneficial effects should be re- evaluated over time.

## Introduction

The rapid spread of the coronavirus disease 2019 (COVID-19) (1) a new highly diffusive disease affecting millions of people, forced the establishment of effective prophylactic and therapeutic approaches. After the first attempts based on existing drugs and convalescent plasma, active and passive immunization strategies, namely vaccination and monoclonal antibodies (mabs), were implemented in just a few months (2) together with new antiviral drugs (3–6). Since the beginning of the pandemic, many people with Inborn Errors of Immunity (IEI) have been infected by SARS-CoV-2 virus (7). These patients showed a different spectrum of disease ranging from asymptomatic to severe with a number of them requiring hospitalization mainly from acute respiratory failure (8). A subgroup of patients with IEI did not respond adequately to COVID-19 vaccinations (9), thus early therapeutic or prophylactic measures were needed to prevent severe or fatal course or COVID-19 and to reduce the burden of hospitalizations (10).

Vulnerable patients such as those with hematological, oncological and respiratory diseases, and immunocompromised patients including those with Inborn Errors of Immunity (IEI) had the chance to be treated with mabs because of significant risk for severe COVID-19. Different experiences associated with positive outcomes in terms of hospitalization and mortality have been reported in COVID-19 fragile categories, such as kidney transplant recipients and hematological patients (11–13).

Up to now, data on effects of COVID-19 treatments in IEI patients have been described (14–16). Recently, our group demonstrated that, during the second year of the pandemic, mabs administration had a beneficial effect on the likelihood of hospitalization and development of severe disease confirming previous data observed in a lower number of patients (17, 18). Poor data in IEI are available regarding antiviral therapy, except for some case reports describing remdesivir as effective for viral clearance in XLA patients and in severely immunocompromised patients (19, 20).

We expanded our previous experience by a multicenter, real life longitudinal study with the aim to evaluate the impact of mabs administration and antiviral treatment on the risk of mortality, hospital admission, and severe disease. As a secondary endpoint, we evaluated the safety of mabs and antiviral drugs in IEI.

## Materials and methods

### Study population

A cohort of 507 patients with IEI in follow-up at four Italian Referral Hospitals for PID (Rome, Naples, Padua, and Cagliari) were included in the study. One hundred and ninety-two experienced SARS-CoV-2 infection between February 1st, 2020 and May 15, 2022. SARS-CoV-2 infection was confirmed by rt-PCR by nasopharyngeal swab. All patients were tested every time they attended a hospital site, in an inpatient and outpatient setting, or when a positive familiar contact was identified irrespective of symptoms, and upon onset of symptoms possibly related to COVID-19. To evaluate the duration of the viral shedding, the dates of the first positive and first negative nasopharyngeal swab were recorded. According to Italian rules in the management of SARS-CoV-2 infected patients, our patients underwent nasopharyngeal swabs 7 days after the first positive swab and then every 3-7 days until a negative one was obtained. In hospitalized patients, nasopharyngeal swabs were performed every three days. From March 2021, patients underwent SARS-CoV-2 immunization by mRNA vaccines. During the study time, patients continued their therapies, including immunoglobulin replacement treatment. Data including type of underlying IEI, sex, age, comorbidities, and ongoing therapies, coronavirus 19 disease severity, hospitalization, vaccination status, and SARS-CoV-2 specific treatments were recorded. COVID-19 severity was defined according to WHO classification (21). Genotype assessment was not systematically conducted. Reinfection was defined as the positive first RT-PCR occurring >120 days after the resolution of the first SARS-CoV-2 infection. The study was approved by the Ethical Committee of the Sapienza University of Rome (CE 4604, n. 316/2016) and was performed in accordance with the Good Clinical Practice guidelines, the International Conference on Harmonization guidelines, and the most recent version of the Declaration of Helsinki.

### Study periods

The long observation period was sequentially analyzed according to the different circulating SARS-CoV-2 strains. In Italy, the original Wuhan strain was first isolated on 21 February 2020 (February to December 2020: wave 1). The SARS-CoV-2 B.1.1.7 (Alpha) variant was first isolated in December 2020 (January 2021 to mid-July 2021: wave 2). Starting from mid-July the B.1.617.2 (Delta) variant was predominant in Italy (July 2021 to December 2021: wave 3). At the end of December 2021, the B.1.1.529 (BA.1), Omicron variant became largely predominant, being replaced by BA.2 in March 2022 (January 2022-up to now: wave 4) (22).

### Treatments

#### Monoclonal antibodies

From March 2021 until December 2021, the monoclonal antibody bamlanivimab (LY-CoV555) or the combinations mabs casirivimab/imdevimab (REGN-CoV-2) and bamlanivimab/etesivimab were administered (23, 24). Starting from December 2021, Sotrovimab has been available (25, 25).

Mechanism of action and approval time of available monoclonal antibodies and antivirals are summarized in **Table 1**. Inclusion criteria for mabs administration are reported in **Supplementary Materials**. Bamlanivimab, the first anti-SARS-CoV-2 mAb targeting the surface spike glycoprotein of the virus, authorized for mild to moderate COVID-19, was related to reduction of hospitalization but authorization was later revoked for single use and a combination therapy with etesevimab was formulated (26–28). Casirivimab/imdevimab combination treatment, authorized for mild to moderate COVID-19, has been found to reduce the risk of any-cause death and viral load more rapidly than placebo. Clinical trials in hospitalized patients, both for Casirivimab/imdevimab and Bamlanivimab/etesevimab, did not show significant differences in survival, symptom alleviation, hospitalization time, or side effects (29, 30). Conversely, in a real-life population, in a pre-Omicron scenario, Casirivimab/imdevimab in comparison to Bamlanivimab/etesevimab was associated with a lower risk of COVID-19 related-hospitalization and death from any cause (31).

**Table 1:** Mechanism of action of mabs and antivirals.

SARS-CoV-2 mabs	Approval date	Mechanism of action
Bamlanivimab	March 2021	Binds to RDB of the spike protein of SARS-CoV-2 and prevents the attachment with the human ACE2 receptor
Bamlanivimab/etesevimab	February 2021	Binds to different but overlapping sites on the RDB, blocking its attachment to the human ACE2 receptor
Casirivimab/imdevimab	February 2021	Binds to non-overlapping epitopes of the spike protein RBB of SARS-CoV-2, blocking ACE2 receptor binding
Sotrovimab	August 2021	RDB binding; inhibits an undefined step that occurs after virus attachment and before fusion of the viral and cell membranes
**Antivirals**	**Approval date**	**Mechanism of action**
Lopinavir/ritonavir	February 2020	Inhibition of papain-like protease (PLpro) e main protease (Mpro), preventing viral replication (off-label use)
Darunavir/ritonavir	February 2020	Inhibition of PLpro e Mpro, preventing viral replication (off-label use)
Remdesivir	February 2021	Stalling RNA-dependent RNA-polymerase (RdRp) causing chain termination of newly formed RNA strain
Nirmatrelvir/ritonavir	December 2021	Peptidomimetic inhibitor of Mpro, rendering it incapable of processing polyprotein precursors, preventing viral replication
Molnupiravir	November 2021	Interaction with RdRp, inducing mutagenesis in viral RNA

Since November 2020, SARS-CoV-2 has started to mutate more drastically, with the simultaneous appearance of a plethora of SARS-CoV-2 variants of concern (VOCs) or variants of interest (VOIs) (23, 32, 33). After the appearance of the Omicron variant, casirivimab-imdevimab and etesevimab-bamlavimab lost their neutralizing activity while Sotrovimab, a new mAb approved in July 2021 in Italy, retained its activity, being effective in reducing mortality and hospitalization in high-risk patients (25, 34, 35).

#### Anti-virals

In March 2020, lopinavir/ritonavir and darunavir/ritonavir became available in Italy as antiviral agents. In October 2020, remdesivir was approved for hospitalized patients (36). From November 2021, new antiviral drugs, such as molnupiravir and PF-07321332/ritonavir respectively, were available. Molnupiravir and nirmatrelvir/ritonavir (Paxlovid) have been approved for use within 5 days from confirmed diagnosis for adult patients at high risk of progression to severe illness (5). Both drugs approved for home treatment were shown to reduce the risk of hospitalization or death from any cause if compared with placebo. Later, also remdesivir was demonstrated to be effective in non-hospitalized patients (37). Inclusion criteria for anti-virals administration are reported in **Supplementary Materials**.

**Figure 1:**
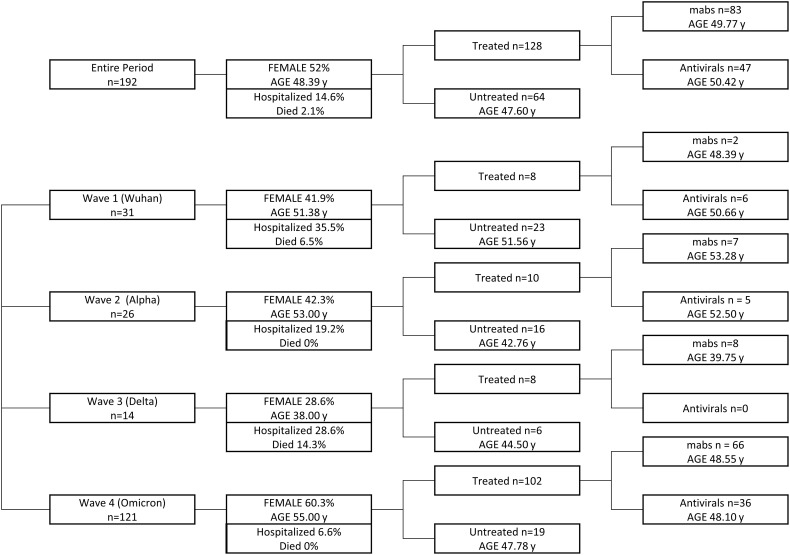
Graphical design of the study. Patients infected in the entire period and during each wave are grouped on the basis of the treatment received, taking into account age and sex representation for each group.

In this real-life study, patients were not randomized to receive either treatment or no treatment at all. Different treatments were administered according to availability at the time of infection. The group of untreated patients is composed of patients who refused treatment for different personal concerns or patients not treated because treatments were unavailable at that specific time. (**Figure 1**).

### Statistical Analysis

Patient characteristics were summarized using medians, standard deviations, ranges, and percentages as appropriate. Chi-squared tests of independence and Fisher’s exact tests were used for categorical data. Mann-Whitney U and Kruskal-Wallis tests were used for unpaired continuous data.

Binomial logistic regression models were fitted to calculate odds ratios (OR) with 95% confidence intervals (CI) for the need of hospitalization and the presence of severe disease in association with mabs or antiviral administration. Multivariable logistic regression analysis was then performed, to confirm the findings, taking into consideration age, sex, co-morbidities and type of IEI as covariates. Statistical significance was considered as a two-tailed *p<*0.05. All the analyses were performed using IBM SPSS statistics 27.0.

## Results

### Descriptive data of COVID-19 course and treatment at first infection

During the study time, a total of 192/507 (37.86%) IEI patients (100 females) were infected with SARS-CoV-2. The cohort of SARS-CoV-2 positive patients included 186 adults and 6 pediatric patients with a median age of 48.39 years (range 10 - 82 years). According to underlying IEI types, 166/452 (36.72%) Common Variable immunodeficiency (CVID), 15/31 (48.38%) X-linked (XLA) or autosomal recessive agammaglobulinemia (ARA), 7/14 patients with Good’s Syndrome (50%), 3/9 (33.33%) patients with Hyper-IgE disease, 1/2 patients with IFNγR deficiency (50%) were SARS-CoV-2 infected. In the whole cohort of infected patients the median duration of rt-PCR positivity was 18 days (IQR 10-27 days). The mortality at first infection across all study periods was 4/192, (2.083%). Overall, 24 patients showed a respiratory exacerbation related to a bacterial infection during COVID-19. Eighteen of 24 patients had pre-existing pulmonary comorbidities such as bronchiectasis (18), granulomatous lymphocytic interstitial lung disease (GLILD) (8) and/or end stage lung disease (4). Twenty-one of 24 were CVID, two were affected by Good’s syndrome and one by ARA. Moreover, 8 of 35 (22.9%) patients with a history of autoimmune (AI) cytopenia developed a recurrence of AI cytopenias, Idiopathic Thrombocytopenic Purpura (ITP) or autoimmune hemolytic anemia, during or soon after the infection. All patients who developed AI recurrence had CVID.

#### Wave 1

From February to December 2020, we reported 31 infected patients: 26, CVID; 3, XLA/ARA; 2, Good’s syndrome. Eighteen patients (58%) were asymptomatic/mild, 4 (12.9%) moderate e 9 (29.03%) severe. Two CVID patients died. Eleven patients were hospitalized. Six patients (19.35%) received inpatient antiviral treatment: 2 were treated with remdesivir, 2 with Lopinavir/Ritonavir, 2 with darunavir/ritonavir. During hospitalization, the two patients who received remdesivir were also treated with bamlanivimab and casirivimab+imdevimab, respectively, for the worsening of their clinical conditions. One patient was treated with hyperimmune plasma and one patient was treated with tocilizumab. The median duration of positive swab was 23 days (IQR 15.5 - 41.5 days).

#### Wave 2

From January 2021 to mid-July we recorded 26 infected patients: 22, CVID; 2, XLA/ARA; 2, Good’s syndrome. Twenty patients were asymptomatic/mild (76.92%), 5 moderate (19.23%) and 1 severe (3.84%). None died. Five patients were hospitalized. Five patients (19.23%) received antiviral treatment: 5 patients received remdesevir. Seven patients (26.92%) were treated with mabs: 3 bamlanivimab, 2 bamlanivimab/etesevimab, 2 casirivimab/imdevimab. A total of three patients received hyperimmune plasma, two of them had already been treated with remdesivir. The median duration of positive swab was 22 days (IQR 18.75 - 27.50 days).

#### Wave 3

During the circulation of B.1.617.2 (Delta) variant we observed a total of 14 infected patients: 12, CVID; 2, XLA/ARA. Ten patients were asymptomatic/mild (71.42%), 1 moderate (7.14%) e 3 severe (21.42%). Two patients died, one CVID and one XLA. Four patients were hospitalized. Eight patients (57.14%) were treated with mabs: 4 patients received casirivimab + imdevimab and 4 received bamlanivimab + etesevimab. At the time of infection, only two patients had received the first dose of vaccine. The median duration of positive swab was 17 days (IQR 9.75-20.00 days).

#### Wave 4

With the spread of Omicron variant a total of 121 patients were infected: 106, CVID; 8, XLA/ARA; 3, Good’s syndrome; 3, Hyper-IgE syndrome; 1, Deficit IFNγR. One hundred and twelve were asymptomatic/mild (92.56%), 8 moderate (6.61%) and one (0.82%) severe. Eight patients were hospitalized. A total of 36 patients (29.75%) were treated with antivirals, 33/36 received their therapy at home. In particular, 5 patients received remdesivir, 26 patients received nirmatrelvir/ritonavir and 5 patients received molnupiravir. A total of 66 patients (54.54%) received mabs: 3 patients bamlanivimab + etesevimab, 9 patients casirivimab+imdevimab, 54 patients Sotrovimab. Six out of 66 were treated with mabs during hospitalization. In 4 hospitalized patients, a combination therapy including antiviral and mabs was administered. 114/121 had received immunization at infection time: one patient had received one dose, 8 patients were immunized with 2 doses, 99 patients had completed 3 doses of vaccine, and 6 patients had received a total of 4 doses. The median duration of positive swab was 14.00 days (IQR 10.00-25.00 days).

Clinical data, hospitalization, and duration of swab positivity on IEI infected patients in relation to the timing of main circulating strains, to antiviral and mabs treatment are summarized in **Table 2**.

**Table 2:** Summary of data of SARS-CoV-2 infected IEI patients in relation to main circulating strains, administered treatment, hospital admission and duration of positive swab.

Time frame	Main circulating VOC	Number of patients	Number of patients withSevere COVID-19	Medianduration of swab positivity (days)	Number of treated patientsmabs	Number of treated patientsantivirals	Number of treated patientsmabs+antivirals	Hospital admissions	Medianhospitalization (days)
01/02/2020 31/12/2020	Wuhan	31	9	23 (15.5-41.5)	2	6	2	11	30.81 (7-90)
01/01/2021 14/07/2021	Alpha	26	1	22 (18.75-27.5)	7	5	0	5	16.2 (6-34)
15/07/2021 25/12/2021	Delta	14	3	17 (9.75-20.00)	8	0	0	4	56 (5-195)
26/12/2021 15/05/2022	Omicron	121	3	14 (10.00-25.00)	66	36	4	8	18.13 (3-40)

### Impact of treatment on COVID-19 severity, hospital admission and mortality

During the entire study period, infected patients treated with mabs and/or antivirals had a lower risk of COVID-19-related hospitalization (p<0.001), development of severe disease (p< 0.001) and death (p=0.037). When we analyzed the effect of mabs and antiviral agents separately, we could confirm a beneficial effect of mabs on risk of hospitalization (p=0.003), and severe disease (p<0.001), whereas antivirals showed a low impact on the risk of hospitalization (p=0.054) only (**Table 3**).

**Table 3:** Impact of treatment with mabs and antivirals on hospitalization, severity of disease and mortality.

	Any treatment		mabs treatment		antiviral treatment	
	n (%) of treated *vs* n (%) untreated	p*	n (%) of treated *vs* n (%) untreated	p*	n (%) of treated *vs* n (%) untreated	p*
Hospitalization	5 (4.6) *vs* 23 (27.4)	<0.001	4 (5.3) *vs* 24 (20.7)	0.003	1 (3.0) *vs* 27 (17.1)	0.054
Severe disease	0 *vs* 14 (17.0)	<0.001	0 *vs* 14 (12.3)	<0.001	0 *vs* 14 (8.9)	0.135
Mortality	0 *vs* 4 (4.8)	0.037	0 *vs* 4 (3.5)	0.157	0 *vs* 4 (2.6)	1

*Fisher’s exact test.

The binomial logistic regression adjusted for age, sex, performed in the cohort of 192 patients infected during the entire study period allowed to confirm that the treatment with mabs and/or antivirals had a positive effect on reducing the risk of hospitalization (OR 0.12, 95%CI 0.043-0.340, p <0.001). When analyzed separately, the same beneficial effect on hospitalization-risk was evident for mabs treatment (OR 0.140, 95%CI 0.045-0.436, p< 0.001) and for antivirals (OR 0.069, 95%CI 0.009-0.551, p=0.012) (**Table 4**). Conversely, mabs and/or antiviral administration had no significant effect on the occurrence of severe disease and death. Regression models showed that GLILD was a risk factor for hospital admission (OR 4.40, 95%CI 1.51-12.86, p<0.007) whereas end-stage lung disease was a risk factor both for exitus (OR 44.22, 95%CI 3.65-536.1, p=0.003), and disease severity (OR 23.14, 95%CI 3.11-181.74, p = 0.002).

**Table 4:** Logistic regression analysis for hospitalization, impact of home-based treatment with mabs and/or antivirals adjusted for sex, and age during the overall study period, after the appearance of Alpha strain and mabs availability (January 2021) and after Omicron strain became predominant (December 2021).

	All study periodN of patients=192	January 2021-May 2022N of patients=161	December 2021-May 2022N of patients=121
**Hospitalization**	**OR (95% CI)	**OR (95% CI)	**OR (95% CI)
mabs and/or antivirals	0.120 (0.043-0.340) p<0.001	0.142 (0.045-0.563) p< 0.001	0.021 (0.002-0.250) p=0.002
mabs	0.140 (0.045-0.436) p<0.001	0.165 (0.048-0.563) p=0.004	0.021 (0.001-0.313) p=0.005
Antivirals	0.069 (0.009-0.551) p=0.012	0.076 (0.009-0.657) p=0.019	0.021 (0.001-0.459) p=0.014

** OR adjusted for age, sex, and CVID diagnosis.

The analysis of the 121 patients who were infected since December 2021 during the Omicron wave demonstrated that any treatment was associated with a lower hospitalization-risk (OR: 0.021, 95%CI 0.002-0.250, p=0.002). This positive impact on the risk of hospital admission was confirmed for either type of treatment (mabs: OR: 0.021, 95%CI 0.001-0.313, p = 0.005; antivirals: OR: 0.021, 95%CI 0.001-0.459, p=0.014). Conversely, no positive impact was observed on development of severe disease and mortality. (**Table 4**). A clinical risk factor for hospital admission was represented by an underlying lung involvement related to GLILD (OR: 8.714 95%CI 1.178-64.471, p < 0.034). Logistic regression analysis adjusted for age and sex, demonstrated that mabs were associated to a lower risk of hospitalization only in patients infected with the BA.1 variant (OR 0.075, 95%C.I. 0.008-0.705; p=0.023) in comparison to patients infected with BA.2 strain (p=0.999), underlining that Sotrovimab could have lost efficacy in the current scenario of the spreading of the BA.2 variant. No differences between BA.1 and BA.2 were observed in terms of hospitalization risk after home-based antiviral treatment

**Figure 2:**
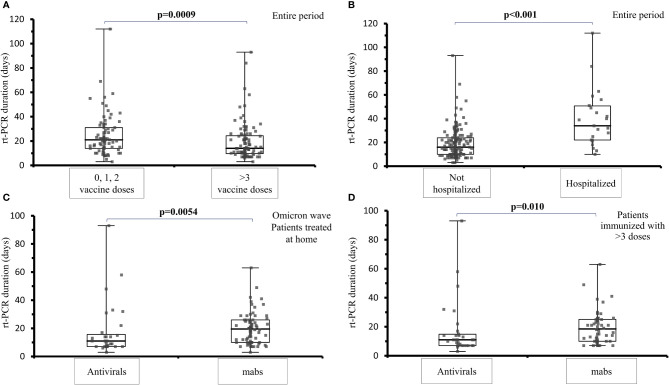
Analysis of viral shedding (SARS-CoV-2 rt-PCR in naso-pharyngeal swab) duration in days in different subsets of IEI patients. **(A)** viral shedding duration at first infection among IEI patients not vaccinated or immunized with 1 or 2 mRNA vaccine doses and IEI patients who had received 3 or 4 vaccine doses, during the entire examined period (median: 14 days, IQR 9.7-24.3 days *vs* 21 days, IQR 14-31 days; p=0.0009). **(B)** comparison of the viral shedding duration in IEI hospitalized (median: 34 days; IQR 22-51 days) and non-hospitalized patients (median: 16 days; IQR 10-23.75 days, p < 0.001), during the entire examined period. **(C)** comparison of the viral shedding duration among IEI patients treated at home with mabs or antivirals at the first infection, during the Omicron wave (median 11 days, range 7-15.7 *vs* 19.5 range 7-26 days, p=0.0054) **(D)** comparison of viral shedding in patients vaccinated with at least 3 doses receiving treatment with antivirals or mabs (11.0 days, IQR 7.0-14.8 *vs* 18.5 days, IQR 10.0-25.1; p=0.010).

During the Omicron wave, the viral shedding was shorter in patients who were treated with antivirals compared to those treated with mabs (median 11 days, range 7-15.7 *vs* 19.5 range 7-26 days, p=0.0054), (**Figure 2C**). The analysis of the viral shedding duration in relation to vaccination status at first infection showed that patients who were immunized with 3 or 4 mRNA vaccine doses had a shorter swab positivity in comparison to patients not immunized or immunized with 1 or 2 doses (median: 14 days, IQR 9.7-24.3 days *vs* 21 days, IQR 14-31 days; p=0.0009) (**Figure 2A**). When considering the sub-cohort of patients who received at least 3 doses of mRNA vaccine, antiviral treatment was still associated with a shorter swab positivity when compared to mabs treatment (11.0 days, IQR 7.0-14.8 *vs* 18.5 days, IQR 10.0-25.1; p=0.010) (**Figure 2D**). Hospitalized patients treated with mabs had a lower risk of ICU admission (0 *vs* 38.9%, p = 0.030) as well as those treated with antivirals (0 *vs* 38.9%, p = 0.030). Viral shedding was longer in hospitalized (median: 34 days; IQR 22-51 days) compared to non-hospitalized patients (median: 16 days; IQR 10-23.75 days, p < 0.001) (**Figure 2B**).

### Re-infection

We registered a total of 16 re-infections (16/192, 8.3%) in 16 patients after a median of 504 days (IQR 394-539) from the first infection. At the time of reinfection, 8 patients had completed 3 doses of immunization, 6 patients had received 2 doses and two patients had refused immunization. Four patients were treated with nirmatrelvir/ritonavir. A total of 9/16 patients were treated with mabs: seven with sotrovimab and two with casirivimab + imdevimab. Two patients were hospitalized during re-infection. One patient who was affected by ARA with a pre-existing end-stage lung disease developed a severe COVID-19 at reinfection and, although treated with casirivimab + imdevimab, he died during hospitalization. The duration of the rt-PCR positivity at the second infection was significantly reduced (median: 14 days, IQR 8.8-16.8 days) in comparison to the median duration of the rt-PCR at their first infection (median: 23 days, IQR 18.2-38.8, p=0.0003). Moreover, duration of rt-PCR positivity was shorter in those treated with mabs (median: 8 days, IQR 8-13.8 *vs* 16 days, IQR 14-18.67, p=0.0041) while it was not different by comparing gender, vaccination status with 3 doses, and use of antivirals (p=0.75, 0.28, and 0.07 respectively).

### Pediatric population

Pediatric population accounted for 6 patients (3 F) with a spectrum of IEI including XLA (3), CVID (2) and HyperIgE syndrome (1) and with a median age of 13 years (range 10 - 16 years). The median duration of rt-PCR positivity was 11.5 days (range 3-29 days). One XLA patient got infected in December 2021. The other five patients had SARS-CoV-2 infection more recently (January-April 2022), possibly related to Omicron spread. All of the patients had a mild COVID-19 with the exception of one recently infected XLA patient not immunized who developed severe COVID-19. During hospitalization he received both remdesivir and Sotrovimab because of worsening of his respiratory condition.

### Adverse events

We did not observe adverse events (AE) both for antiviral and mabs in the vast majority of patients.

Only one CVID patient hospitalized for pneumonia experienced fever and chest pain shortly after the infusion of casirivimab/imdevimab. The same patient developed an ITP exacerbation. No AE were reported by patients receiving antiviral treatments.

## Discussion

Public health measures and vaccination campaigns of many extremely vulnerable individuals to SARS-CoV-2 have been implemented during the first years of the pandemic (31, 38–40). Age, sex, high BMI, diabetes, renal and liver impairment, hemoglobinopathies, neurodegenerative diseases, cardiovascular and pulmonary co-morbidities, as well as immunocompromission, have been associated with hospitalization and severe course of COVID-19 both in adolescents and adult patients, thus leading to prioritization for specific early treatment (41–43). In real-world studies, early treatment (within 5 days of symptom onset) with monoclonal antibodies or antivirals was associated with significant reduction in hospital admission, severe disease and hospitalization (11–13, 15, 28–31).

SARS-CoV-2 infected IEI patients represent potential candidates for passive immunization, considering that the rate of response to vaccination is still unclear but overall reduced (ranging 33% to 70%), and that clinical trials of passive immunoprophylaxis have shown promising results in subjects at increased risk of an inadequate response to vaccination (9, 10, 44, 45). Indeed, the type and duration of protection after infection and/or vaccination is still unknown, also in the context of emergence of new variants (19). Moreover, despite presenting relatively young age and consequently lower prevalence of cardiovascular disease, IEI patients may be affected by several other comorbidities related to the underlying immunodeficiency (46).

The analysis of our cohort, across 26 months, showed the efficacy of antivirals on the risk of hospitalization, while mabs may have offered a positive effect on hospitalization, and COVID-19 severity. In addition, hospitalized patients treated with mabs and antivirals had a reduced risk of ICU admission. This protection was consistent across the alpha, delta and early omicron waves, although the emergence of BA.2 probably reduced the effect of available mabs. Accordingly, impaired viral neutralization and reduced clinical efficacy of sotrovimab is being reported (47), leading to withdrawal of authorization by FDA in U.S. areas where BA.2 was the prevalent strain (48). On the other hand, recent reports suggest that remdesivir, molnupiravir and nirmatrelvir-ritonavir may retain their efficacy against omicron BA.2 (5, 49).

Our results regarding the impact of mabs and antivirals in IEI patients during the Omicron BA.1 and BA.2 period are in line with these findings. Indeed, home-based antiviral treatment was still able to reduce the time of rt-PCR positivity, also in patients immunized with at least 3 doses, when compared to mabs.

The analysis of the viral shedding duration in relation to vaccination status at first infection shows that patients who were immunized with 3 or 4 mRNA vaccine doses had a shorter positivity in comparison to patients not immunized or immunized with 1 or 2 doses. Interestingly, a comparison of viral shedding in patients vaccinated with at least 3 doses outlined a shorter period of positivity in patients receiving antivirals in comparison to those who were treated with mabs. This might be due to the effect of the booster dose, particularly in terms of cellular immunity (50), together with a beneficial additive effect of antivirals. The poor effect of the combination of immunization and mabs treatment on the time of viral shedding might underlie the lack of mucosal immunity protection in particular in IEI patients with predominant antibody defects.

Our data supports the observation that non-infectious comorbidities worsened the course of COVID-19. In fact, during the whole period, a pre-existing end stage lung disease was found to be related with higher risk of severe disease, ICU admission and death (46, 51). In particular, across the different waves, including the ongoing Omicron, a risk factor for hospital admission was represented by an underlying GLILD. This may be important, considering the increased risk of superinfections, VAP and other potential complications that may arise in IEI patients admitted to ICU.

We here show that reinfection is becoming a non-infrequent event among IEI, similarly to the general population. Up to now, the number of described cases of reinfections in IEI is limited to a few case reports involving XLA patients, characterized by a deep impairment of B cell function. Moreover, these reports described reinfections in patients with IEI only in the pre-omicron period (52). In the present study, reinfections have been mostly reported during Omicron spread. The long interval between reinfections was also enough to safely exclude long-term viral persistence. Time of positivity of SARS-CoV-2 rt-PCR at reinfection was shorter among those treated with mabs. Presumably, many factors may have contributed to this result, including the recall response to reinfection and the hybrid immunity determined by vaccination after a previous infection, even in the context of defective mucosal and systemic immune response. However, we cannot determine the relative contribution of each of those factors. Despite available treatments, SARS-CoV-2 reinfection may still lead to hospitalization and death, as well as immune dysregulation leading to autoimmune phenomena; the patient comorbidities, in particular end-stage lung disease, may also play a relevant role.

One of the peculiarities of our study is that it is a real-life cohort study, including all patients with specific IEIs in active follow-up in four referral centers, offering a complete view of all SARS-CoV-2 infected patients, both hospitalized and outpatient, and taking into account early administered treatments. The number of enrolled patients is significant when considering that all the included IEIs are rare diseases. As far as we are aware, only few data are available regarding the same treatments in patients with secondary immunodeficiencies, mainly focusing on severe COVID-19 and hospitalized patients. In the available studies, SARS-CoV-2 specific treatments were often late administered during the disease course (51, 53). Moreover, available series and studies generally group patients according to the cause of secondary immunodeficiencies (e.g. hematological malignancies) rather than on the type of immune defect (26, 27). This makes it harder to understand the relationship between patients’ immune status and impact of specific treatments on COVID-19 course. In these patients, we also have no clear evidence on the actual impact of innate and mucosal immunity on the course of COVID-19. It has been reported that patients with innate immune deficiencies may undergo a more severe COVID-19 course (54). Moreover, mucosal immunity represents the first barrier against SARS-CoV-2 and several factors, both intrinsic (e.g. IgA deficiency) and iatrogenic (e.g. antibiotic prophylaxis and treatments) may impact on its function. IgA, active against several viruses including rotavirus, poliovirus, influenza virus, are able to protect the epithelial barriers from pathogens and modulate excessive immune responses in inflammatory diseases. IgA response is meant to dominate early SARS-CoV-2 specific humoral response, contributing to viral neutralization to a greater extent than IgG. It has indeed been suggested that the lack of anti-SARS-Cov-2 IgA and secretory IgA might explain COVID-19 severity, vaccine failure, prolonged viral shedding and reinfection (55). On the other hand, higher SARS-CoV-2 specific IgG than IgA levels have been reported in the saliva of immune-competent vaccinated patients, and IgG salivary concentration increases in parallel with the increase of specific serum IgG levels (56). We might thus hypothesize that mabs administration may lead to a rapid increase of SARS-CoV-2 specific antibodies in saliva, thus justifying the beneficial effect of mabs also in patients with limited spontaneous IgA production.

Nevertheless, our data show that vaccination with 3 doses has an impact on disease duration, that might be due also to an increased mucosal IgA and/or IgG content in a subgroup of responders and not only to cellular response (56). In this way, development of new vaccines able to induce a stronger IgA response might hopefully strengthen the protection (57). However, due to the emergence of VOCs and the evidence of a reduced neutralizing efficacy of mabs as well as natural antibodies (58) therapeutic strategies based on the use of antivirals might be impaired to a lesser extent by SARS-CoV-2 “escape mechanisms”.

The low rate of adverse effects observed in our cohort, after mabs and antivirals administration, is in line with data from literature showing - in a real-world evaluation - 1.2% monoclonal antibody infusion-associated adverse events, with 0.3% only considered as severe (59). In a recent meta-analysis, all mabs demonstrated good safety profiles with no significant increase in adverse events although Casirivimab + imdevimab and sotrovimab were associated with significant reductions in the odds of serious adverse events, with sotrovimab ranking first and casirivimab + imdevimab ranking second in terms of safety (60). The available evidence of AE is scant regarding antivirals, especially those for at-home therapy, but an incidence comparable with the placebo arm has been reported (61).

The main limitations of our work are the lack of systematic SARS-CoV-2 strain typization in individual patients and the small sample of patients treated with antivirals. Thus, the mabs and antiviral clinical beneficial effect data should not be considered as definitive proof of a given single drug efficacy. On the other hand, in such rare diseases it is not feasible to produce strong evidence as for patients with cardiovascular co-morbidities or secondary immunodeficiencies.

Further studies are needed to specifically address the optimal treatment strategy in IEI infected by SARS-CoV-2. In our cohort, mortality for COVID-19 at first infection during the pandemics was around 2.1%, higher than in the general Italian population, even if not statistically different. The widespread use of specific therapies, vaccination and better access to care might have contributed to mitigate this figure. However, the rapid spread of new viral strains underlines that mabs and antiviral beneficial effects should be re-evaluated over time.

## Data availability statement

The raw data supporting the conclusions of this article will be made available by the authors, without undue reservation.

## Author contributions

CM, FC, GG, DF, and IQ conceptualized the study. FC, GG, DF, MR, GS, IQ, and CM designed the protocol study. GDN, GL, AP, PB, BLC, GC, RS, and FP recruited patients and collected data. DF and FC did the statistical analysis. GG, FC, DF, IQ, and CM prepared the first draft of the manuscript. All authors reviewed the manuscript before publication. All authors contributed to the article and approved the submitted version.

## Conflict of interest

The authors declare that the research was conducted in the absence of any commercial or financial relationships that could be construed as a potential conflict of interest.

## Publisher’s note

All claims expressed in this article are solely those of the authors and do not necessarily represent those of their affiliated organizations, or those of the publisher, the editors and the reviewers. Any product that may be evaluated in this article, or claim that may be made by its manufacturer, is not guaranteed or endorsed by the publisher.

## Abbreviations

AEs, Adverse Events; AI, Autoimmune; AIFA, Agenzia Italiana del farmaco; ARA, Autosomal Recessive Agammaglobulinemia; CI, confidence interval; COVID-19, CoronaVirus Disease-2019; CVID, Common Variable Immunodeficiency; FDA, Food and Drug Administration; GLILD, Granulomatous Lymphocytic Interstitial Lung Disease; ICU, Intensive Care Unit; IEI, Inborn Errors of Immunity; IFNγR, γ-Interferon Receptor; IQR, Interquartile range; ITP, Idiopathic Thrombocytopenic Purpura; mabs, Monoclonal Antibodies; mRNA, Messenger RiboNucleic Acid; OR, Odds Ratio; PID, Primary Immunodeficiency; Rt-PCR, Real-time Polymerase Chain Reaction; SARS-CoV-2, Severe Acute Respiratory Syndrome Coronavirus 2; VAP, Ventilator-Associated Pneumonia; VOCs, Variants of Concern; VOIs, Variants of interest; XLA, X-linked Agammaglobulinemia; WHO, World Health Organization.
