# Supplementary material for: Real-life data on monoclonal antibodies and antiviral drugs in Italian inborn errors of immunity patients during COVID-19 pandemic
Source: Front Immunol. 2022 Jul 28;13:947174. doi: 10.3389/fimmu.2022.947174 (PMC9367468; doi:10.3389/fimmu.2022.947174)
Supplement: Supplementary file 1 [file DataSheet_1.pdf]

Supplementary Methods

Inclusion Criteria for mAbs treatment

Since the advent of mAb therapy in Italy, on March 2021, patients were candidate when meet the following criteria: laboratory-confirmed SARS-CoV-2 infection < 5 days, symptom of mild to moderate COVID-19 evolving for ≤ 10 days, and at least one of following risk factors for progression to severe disease: 65 years and older, body mass index (BMI) >30, any chronic renal impairment (including subjects undergoing peritoneal dialysis or hemodialysis), uncontrolled or complicated diabetes mellitus, any immunocompromising condition (including primary and secondary immunodeficiencies), cardio-cerebrovascular diseases (including hypertension with concomitant organ damage), COPD or other chronic respiratory diseases, chronic liver disease, hemoglobinopathies, neurodevelopmental and neurodegenerative disease. Patients requiring hospitalization for COVID-19 or supplemental oxygen therapy were excluded. (*Determina AIFA Nella GU N.142 Del 16.06.2021*. Available at: https://www.gazzettaufficiale.it/eli/gu/2021/06/16/142/sg/pdf.

Inclusion Criteria for antiviral treatment

From November 2021 AIFA approved the use of oral antivirals, such as molnupiravir (Lagevrio) and PF-07321332/ritonavir (Paxlovid).

Paxlovid is indicated for the treatment of COVID-19 in adults who do not require supplemental oxygen therapy and who are at high risk of progression to severe COVID-19 (Determina AIFA DG 160/2022 del_31.01.2022)

Molnupiravir is used for the treatment of coronavirus disease 2019 in non-hospitalized adult patients with recent mild to moderate illness onset and with concomitant clinical conditions that represent specific risk factors for the development of severe COVID-19. (Determina AIFA DG/1644/2021, December 2021).

In addiction to oral antiviral drugs, Remdesivir (approved from February 2021 in Italy) is given to treat COVID-19 in:

• adults and adolescents (aged 12 to less than 18 years who weigh at least 40 kg) with pneumonia and who need supplemental oxygen therapy to breathe, but who are not receiving artificial ventilation (which involves the use of mechanical means to assist or replace spontaneous breathing at the start of treatment);

• adults who do not need supplemental oxygen therapy to breathe and who have an increased risk of progressing to severe COVID-19.

(Determina AIFA/92/2020 del 8/10/2020)

Severe COVID-19 risk progression factors are the same defined for mAbs.
